# Tensile Mechanical Properties of Dry Cortical Bone Extracellular Matrix: A Comparison Among Two Osteogenesis Imperfecta and One Healthy Control Iliac Crest Biopsies

**DOI:** 10.1002/jbm4.10826

**Published:** 2023-10-11

**Authors:** Michael Indermaur, Daniele Casari, Tatiana Kochetkova, Bettina M. Willie, Johann Michler, Jakob Schwiedrzik, Philippe Zysset

**Affiliations:** ^1^ ARTORG Center for Biomedical Engineering University of Bern Bern Switzerland; ^2^ Swiss Federal Laboratories for Material Science and Technology Thun Switzerland; ^3^ Research Centre, Shriners Hospital for Children‐Canada, Department of Pediatric Surgery McGill University Montreal QC Canada

**Keywords:** MICRO TENSILE EXPERIMENT, NANOINDENTATION, OSTEOGENESIS IMPERFECTA, QUANTITATIVE RAMAN SPECTROSCOPY

## Abstract

Osteogenesis imperfecta (OI) is a genetic, collagen‐related bone disease that increases the incidence of bone fractures. Still, the origin of this brittle mechanical behavior remains unclear. The extracellular matrix (ECM) of OI bone exhibits a higher degree of bone mineralization (DBM), whereas compressive mechanical properties at the ECM level do not appear to be inferior to healthy bone. However, it is unknown if collagen defects alter ECM tensile properties. This study aims to quantify the tensile properties of healthy and OI bone ECM. In three transiliac biopsies (healthy *n* = 1, OI type I *n* = 1, OI type III *n* = 1), 23 microtensile specimens (gauge dimensions 10 × 5 × 2 μm^3^) were manufactured and loaded quasi‐statically under tension in vacuum condition. The resulting loading modulus and ultimate strength were extracted. Interestingly, tensile properties in OI bone ECM were not inferior compared to controls. All specimens revealed a brittle failure behavior. Fracture surfaces were graded according to their mineralized collagen fibers (MCF) orientation into axial, mixed, and transversal fracture surface types (FST). Furthermore, tissue mineral density (TMD) of the biopsy cortices was extracted from micro–computed tomogra[hy (μCT) images. Both FST and TMD are significant factors to predict loading modulus and ultimate strength with an adjusted *R* ^2^ of 0.556 (*p* = 2.65e−05) and 0.46 (*p* = 2.2e−04), respectively. The influence of MCF orientation and DBM on the mechanical properties of the neighboring ECM was further verified with quantitative polarized Raman spectroscopy (qPRS) and site‐matched nanoindentation. MCF orientation and DBM were extracted from the qPRS spectrum, and a second mechanical model was developed to predict the indentation modulus with MCF orientation and DBM (*R* ^2^ = 67.4%, *p* = 7.73e−07). The tensile mechanical properties of the cortical bone ECM of two OI iliac crest biopsies are not lower than the one from a healthy and are primarily dependent on MCF orientation and DBM. © 2023 The Authors. *JBMR Plus* published by Wiley Periodicals LLC on behalf of American Society for Bone and Mineral Research.

## Graphical abstract

This study compares the micro tensile mechanical properties of dry cortical bone extracellular matrix among two osteogenesis imperfecta and one healthy control biopsies.

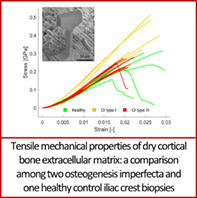

## Introduction

Osteogenesis imperfecta (OI), also known as “brittle bone disease”, is a collective term of genetic bone disorders. Most cases of OI are triggered by mutations in genes encoding type I collagen (COL1A1 and COL1A2). OI can be classified into different severity types. OI type I is the mildest and OI type III the most nonlethal severe form.^[^ ^1^ ^]^ Individuals with OI have a high rate of bone fractures, especially during growth.^[^ ^2^ ^]^ Thus, it is crucial to understand the contribution of the mechanical properties and structure to bone fragility in individuals with OI.

At the macroscopic scale, OI bone features reduced bone quantity and quality. The areal bone mineral density (aBMD) measured with dual‐energy X‐ray absorptiometry and trabecular bone volumetric mineralization (vBMD) assessed with HR‐pQCT is reduced in persons with OI.^[^ ^3^, ^4^ ^]^ Cortical bone is thinner and includes more pores, whereas cancellous bone has thinner and fewer trabeculae.^[^ ^5^ ^]^ Furthermore, trabecular bone structure is prone to be more heterogeneous.^[^ ^6^ ^]^ However, the degree of bone mineralization is increased in OI bone compared to healthy control bone measured with various methods (micro–computed tomography [μCT],^[^ ^7^, ^8^ ^]^ Raman spectroscopy,^[^ ^8^ ^)^ or quantitative backscattered electron imaging [qBEI]^[^ ^9^, ^10^ ^]^). In fact, the width of the mineral particles is not different in OI than in healthy control bone, but the mineral particles are more densely packed (12% higher density than control), which leads to a higher level of mineralization in individuals with OI.^[^ ^11^ ^]^ In the last three decades, bisphosphonates (eg, zoledronate, pamidronate), which suppress bone resorption, have been widely used by individuals with OI during childhood and to a lesser extent during adulthood.^[^ ^12^, ^13^ ^]^ Interestingly, it has been reported that bisphosphonate therapy does not alter the mineralization and the indentation properties at the extracellular matrix level (ECM) in children.^[^ ^9^ ^]^ Nevertheless, bisphosphonates decrease the fracture rate in OI individuals, but do not negate fracture risk.^[^ ^14^, ^15^, ^16^ ^]^

Bone is composed of a complex and unique hierarchical structure allowing it to be a stiff material that can dissipate energy with several toughening mechanisms and features different mechanical behavior and interactions at different levels. At the ECM level, various methods exist to extract the mechanical properties. The most established technique is nanoindentation, which measures elastic properties but also hardness and dissipated energy (post‐yield).^[^ ^17^, ^18^, ^19^ ^]^ Recently, methods have been developed that capture the post‐yield behavior at the ECM level, known as micropillar compression^[^ ^20^, ^21^, ^22^ ^]^ and microtensile testing.^[^ ^23^, ^24^ ^]^ All methods revealed high anisotropic mechanical behavior in the elastic and post‐yield phases. The elastic modulus, yield stress, and hardening/softening behavior of bone ECM micropillars significantly depend on the orientation of the mineralized collagen fibers (MCFs).^[^ ^20^, ^25^, ^26^ ^]^ Additionally, the mechanical properties are generally higher at the lamellar level than at the macroscopic level. This size effect can be explained by stress concentrations produced by resorption spaces, lacunar and vascular porosity, defects, microcracks, and interfaces present at the macroscopic level that turns bone into a quasi‐brittle material.^[^ ^20^ ^)^

Only a few studies analyzed the mechanical properties of OI bone at the tissue level. Two of the studies used nanoindentation and found that the indentation modulus is higher in OI bone compared to healthy control,^[^ ^9^, ^27^ ^]^ whereas another study found opposite results.^[^ ^8^ ^]^ Additional studies reported that elastic modulus and hardness of OI type III bone from a fracture site are decreased compared to OI type I,^[^ ^28^ ^]^ with similar properties found between OI type III and IV.^[^ ^29^ ^]^ A recent study by Indermaur and colleagues^[^ ^27^ ^]^ showed that the post‐yield behavior of OI bone in compression is not inferior compared to healthy control bone and increases with mineralization. They hypothesize that the brittle nature of OI bone compared to healthy bone may be explained by the reduced macroscopic bone quality and quantity and altered tissue properties of OI bone in tension.

Casari and colleagues^[^ ^30^ ^]^ developed a new microtensile setup to quantify the tissue mechanical properties of ovine bone in tension in dry^[^ ^23^ ^]^ and in rehydrated^[^ ^24^ ^]^ conditions at the ECM level of bone. In both states, ovine bone showed an anisotropic elastic modulus and ultimate strength with a size‐effect, meaning that the ultimate strength is higher at the tissue level compared to macroscopic properties. Moreover, the bone matrix in dry conditions shows a brittle behavior while revealing a post‐yield behavior in wet condition. Unfortunately, rehydration of dry bone tissue may lead to swelling, which alters the of testing.^[^ ^24^ ^]^

The goal of this study is to compare the tensile mechanical properties among bone biopsies from individuals with and without OI. Our first hypothesis is that a size‐effect exists between the micro and macro mechanical properties leading to increased bone strength at the tissue level in both OI and healthy control bone. Second, we hypothesize that the mechanical properties depend on the angular orientation of the MCFs and the mineralization in both OI and healthy control bone. The last hypothesis is that the reduced amount and improperly formed collagen type I in OI bone reduces the mechanical properties and reveals a more brittle post‐yield behavior. To test this hypotheses, microtensile experiments are conducted in human bone ECM on two OI and one healthy biopsies. Then, the fracture surfaces are inspected and graded according to their MCF orientation. Additionally, global mineralization is measured using μCT. Next, relationships between the tensile properties, MCF orientation, and global mineralization are analyzed. Finally, the findings are supported by nanoindentation and site‐matched quantitative polarized Raman spectroscopy measurements.

## Materials and Methods

**Fig. 1:**
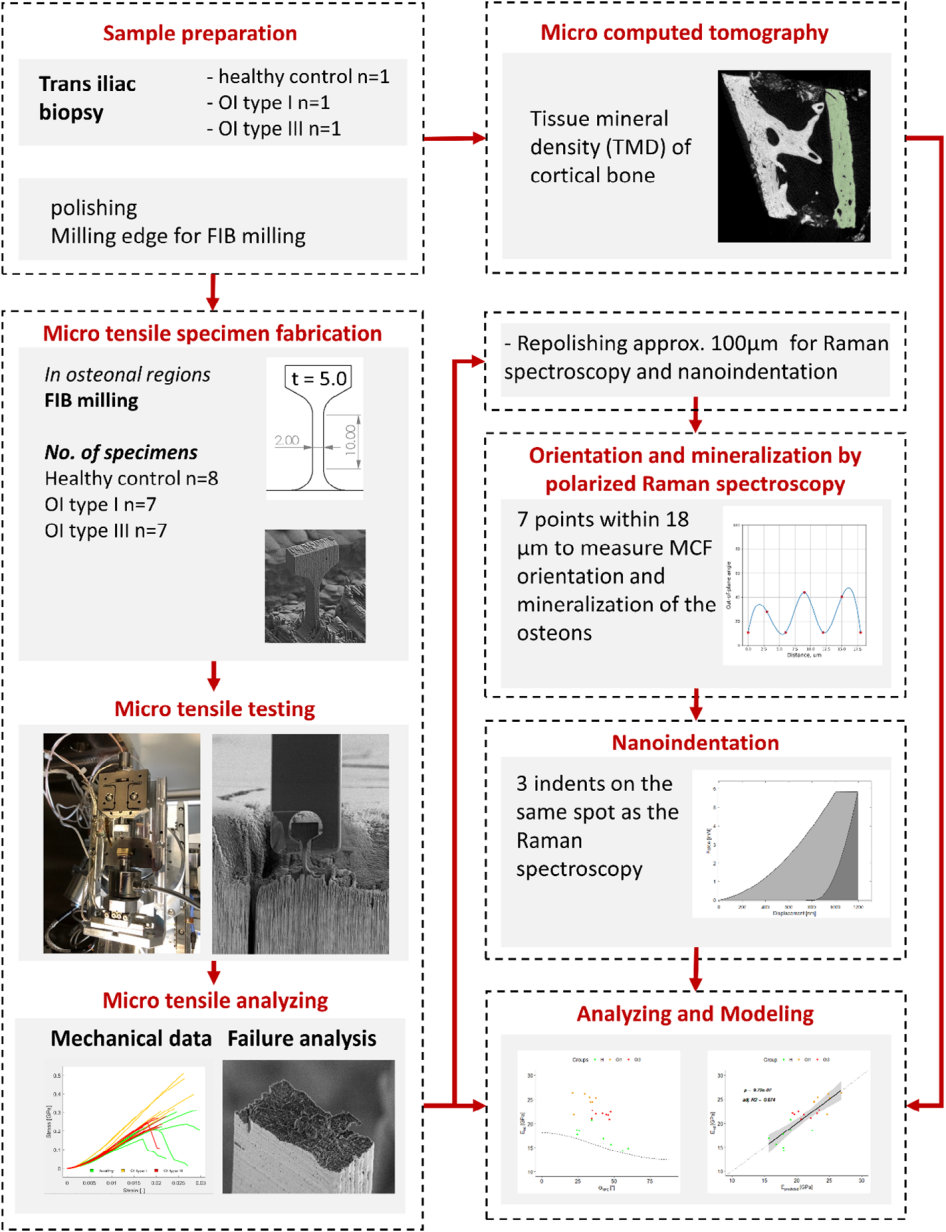
Illustration of the different steps in the study: On three trans iliac biopsies (healthy *n* = 1, OI type I *n* = 1, and OI type III *n* = 1), microtensile experiments were conducted, mechanical properties were quantified, and fracture surfaces were analyzed. Global mineralization was measured using μCT. Additional quantitative polarized Raman spectroscopy (qPRS) and site‐matched nanoindentation measurements were performed. Last, relationships between mechanical properties, mineralized collagen fiber (MCF) orientation, and degree of mineralization were analyzed.

The study can be divided in different phases, which are detailed in Fig. 1.

### Biopsies

The study comprises three transiliac biopsies (healthy control *n* = 1, OI type I *n* = 1, OI type III *n* = 1), which were reused from two previously published studies (healthy/control biopsy of Glorieux and colleagues in 2000^[^ ^31^ ^]^ and OI biopsies of Rauch and colleagues in 2006^[^ ^32^ ^]^). Selection criteria for the individuals and biopsies and the obtaining procedures were reported in the corresponding articles. These studies reported in detail the fixation protocol, which was the same for all three biopsies. Briefly all samples underwent fixation in formalin, dehydration in ethanol, and embedding in polymethylmethacrylate (PMMA). The two OI biopsies were extracted from a 20‐year‐old female with OI type I (mutated gene = COL1A1, bone volume/total volume [BV/TV] of whole biopsy = 20.27%) and a 19‐year‐old female with OI type III (mutated gene = COL1A2, BV/TV of whole biopsy = 7.07%) after receiving bisphosphonate (pamidronate) treatment for approximately 5 years.^[^ ^32^ ^]^ The healthy control specimen was donated by a 20‐year‐old male (BVTV of whole biopsy = 29.69%).^[^ ^31^ ^]^ The three specimens were also included in a previous study, in which the compressive properties of OI bone were analyzed at the ECM level.^[^ ^27^ ^]^

### μCT

The biopsies were scanned using hydroxyapatite calibrated μCT (microCT 100; Scanco Medical AG, Brüttisellen, Switzerland) with a resolution of 10 μm (energy = 45 kVp, tube current = 200 μA, integration time = 300 ms). The cortical bone of the biopsy was isolated in the μCT image, and the tissue mineral density (TMD) was calculated in mg HA/cm^3^ per biopsy (Matlab R2018b; MathWorks, Natick, MA, USA).^[^ ^27^ ^]^

### Microtensile experiment

#### Microtensile specimen fabrication

Microtensile specimens were fabricated with the protocol described by Casari and colleagues.^[^ ^23^ ^]^ Before manufacturing the microtensile specimens, the biopsies had to be preprocessed. The biopsies were lapped (Logitech PM5; Logitech Limited, Glasgow, UK, with a 1000 grit SiC powder) and polished (Logitech PM5) with an ultra‐fine Al_2_O_3_ powder (grain size 0.05 μm) to produce a smooth and flat top surface. Afterward, the remaining polishing particles were removed in an ultrasonic saline solution bath for 60 seconds. To produce the T‐bar‐shaped microtensile specimens using focused ion beam (FIB) milling technique, access needed to be guaranteed from the top surface and perpendicular to it. Therefore, a step of approximately 0.5 mm in height through the cortical wall of the iliac biopsy was trimmed using a conventional milling machine (milling parameters: rotational speed of 2000 RPM, iterative cutting depth of 0.05 mm) to expose the middle of the osteons (see Fig. 2.1‐3). Then, a thin gold film of 10‐nm thickness was sputtered on the biopsy surface in a high vacuum sputter coater (Leica EM ACE600; Leica, Wetzlar, Germany) to reduce potential drift due to charging in the scanning electron microscope (SEM). Finally, the edge was visually inspected inside an SEM (Hitachi S‐4800, Hitachi, Tokyo, Japan) to check for potential cracks created by the conventional milling process.

**Fig. 2:**
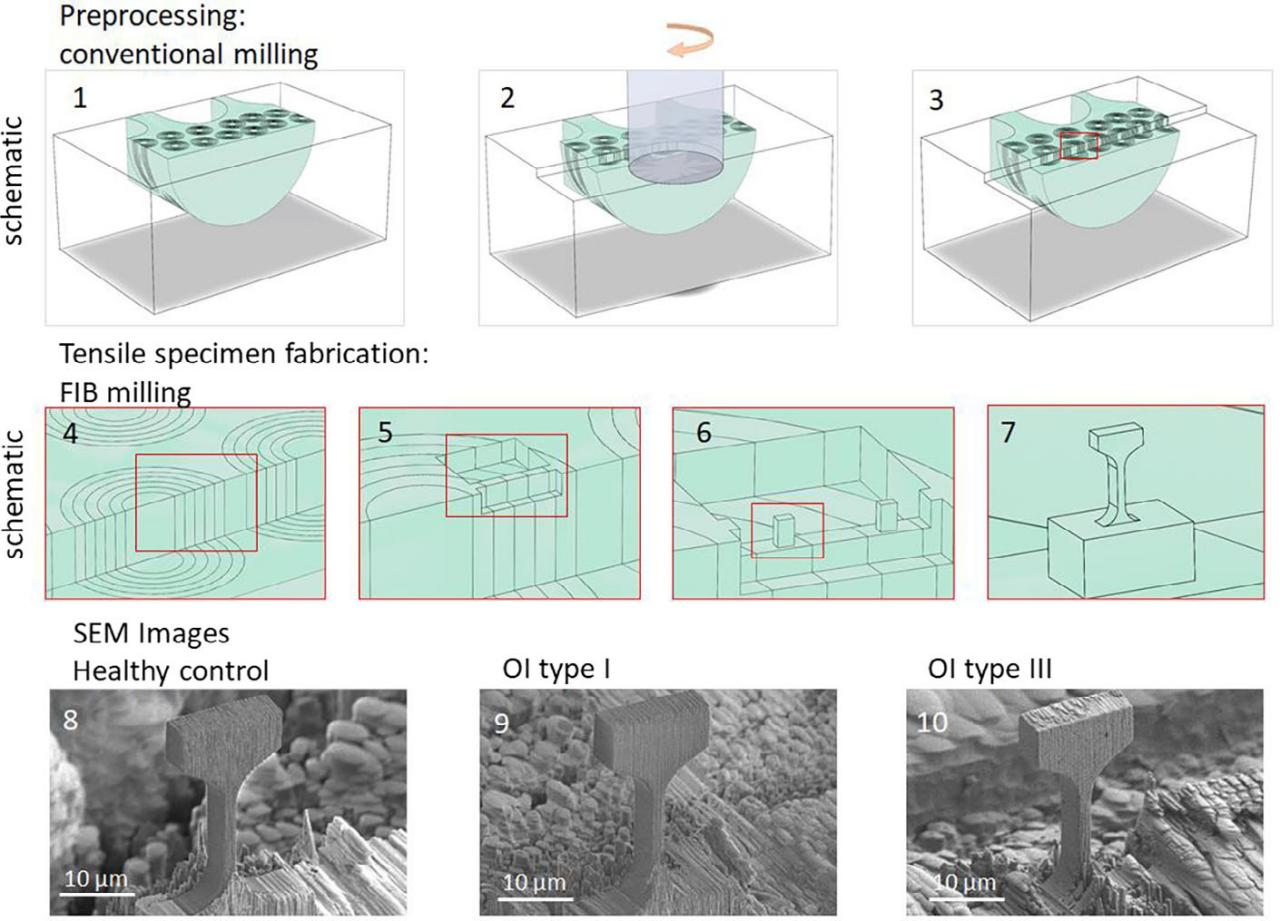
Schematic overview of the microtensile fabrication process. Top row: Preprocessing conventional milling with (*1*) initial trans iliac biopsy embedded in PMMA, (*2*) conventional milling of the step, and (*3*) final state after conventional milling. Middle row: Tensile specimen fabrication using focused ion beam (FIB) milling: (*4*) lamellar structure of the osteon, (*5*) rough cut to produce a wall, (*6*) middle cut to produce battlement‐like structure and (*7*) fine milling to produce the final T‐bar shaped structure. Bottom row: representative scanning electron microscope (SEM) images (*8*) healthy control, (*9*) OI type I, and (*10*) OI type III.

Tensile specimens were fabricated in osteonal regions and a few tens of micrometers away from the edge to avoid potential artifacts due to the former milling procedure. Specimens were fabricated using FIB milling. Initially, 30–40‐μm‐deep trenches were prepared around the region of interest using a Xe plasma‐FIB (Tescan Fera; Tescan, Brno, Czech Republic) operated at 30 kV with beam currents of 600–100 nA. In order to avoid excessive FIB‐induced damage due to this rough milling step, the resulting wall structure (Fig. 2.5) was milled to a thickness of 20 μm (4 times the thickness of the final geometry). Successively, T‐bar‐shaped microtensile specimens featuring a final gauge length of approximately 2 × 5 × 10 μm^3^ were fabricated using a Ga FIB (Tescan Lyra; Tescan, Brno, Czech Republic) operated at 30 kV while sequentially stepping down beam currents from 10 to 0.2 nA. The detailed milling procedure was developed and described by Casari and colleagues.^[^ ^23^ ^]^ A schematic overview of the different steps is shown in Fig. 2 (subfigure 4–7). In total, 23 specimens were fabricated from the three iliac biopsies (healthy control *n* = 8, OI type I *n* = 7, OI type III *n* = 8). The manufacturing time was approximately 6 hours per specimen. Representative microtensile specimen SEM images for each biopsy are shown in Fig. 2 (subfigure 8–10).

#### Microtensile testing

Microtensile specimens were loaded quasi‐statically (5 nm/s) inside an SEM (Tescan Mira; Tescan, Brno, Czech Republic) under vacuum using a microtensile setup (Alemnis, Thun, Switzerland). A customized gripper was used to compensate for potential misalignment.^[^ ^30^ ^]^ During testing, a video was captured, and the reaction force and displacements were recorded (10 Hz). Force and displacement were converted into stress and strain by accounting for the previously measured geometry, according to Casari and colleagues.^[^ ^23^ ^]^ Loading modulus (E_loading_ [GPa]), ultimate stress (σ_ult_ [Mpa]), and strain to failure (ε_ult_ [−]) were extracted. Loading modulus includes the elastic (reversible) and plastic (irreversible) deformation during loading and was defined by the highest slope in the loading face. Brittle materials in tension reveal a small post‐yield deformation, and ultimate stress and strain to failure can therefore be used to describe the maximum strength of such materials.

#### Microtensile fracture surface

After testing, fracture surfaces were inspected using an SEM (S‐4800; Hitachi, Tokyo, Japan). The fracture surfaces were classified (in a randomized survey) by four individual, independent raters according to their primary MCF alignment in respect to the gauge cross‐section. Three fracture surface types (FST) were distinguished: (i) axial, where the MCFs were pulled out of the bone matrix, (ii) transverse, where the failure occurred between the MCFs and (iii) mixed type. Additionally, the presence of canaliculi and other voids on the surface were checked, which may act as potential stress concentrators.

#### Microtensile model

In compression, the mechanical properties of bone are significantly dependent on the alignment of the MCFs^[^ ^20^, ^25^, ^33^ ^]^ and the degree of mineralization.^[^ ^27^ ^)^ A multilinear model for the loading modulus (Eq. 1) and ultimate strength (Eq. 2) was developed to verify this assumption in tension. The model contains the FST, the TMD as factors, global fitted scalars α and β for the FST and TMD, and an error term ϵ combining intercept and the uncertainty. The model allows to test if the FST and TMD are significant contributors of the tensile properties similar in compression. The tensile model got verified with the later described indention model containing local MFC orientation and mineralization. The model was fitted in R (R version 3.6.0; R Foundation for Statistical Computing, Vienna, Austria; https://www.r-project.org/).

(1) $$E_{\text{loading}}=\text{αFST}+\text{βTMD}+\epsilon ,$$

(2) $$\sigma_{\mathrm{ult}}=\text{αFST}+\text{βTMD}+\epsilon ,$$

### Quantitative polarized Raman spectroscopy

Local composition and MCF orientation measurements were performed using quantitative polarized Raman spectroscopy (qPRS, WITec Alpha 300 R; Leica, Ulm, Germany; 785 nm laser wavelength and 30 mW power, 50× objective with 0.8 NA). Unfortunately, collecting site‐matched Raman spectra on the fracture surfaces of the tested microtensile specimens was not possible. The gold on the surrounding bone and remaining Ga− ions of the FIB milling process corrupted and falsified the Raman spectra. Therefore, the top surface of the biopsies was repolished (approximately 100 μm) to remove all gold and implanted gallium ions. The light microscope images of the surface were taken before and after polishing to perform the qPRS in the same osteonal region as those where the microtensile specimens were obtained.

**Fig. 3:**
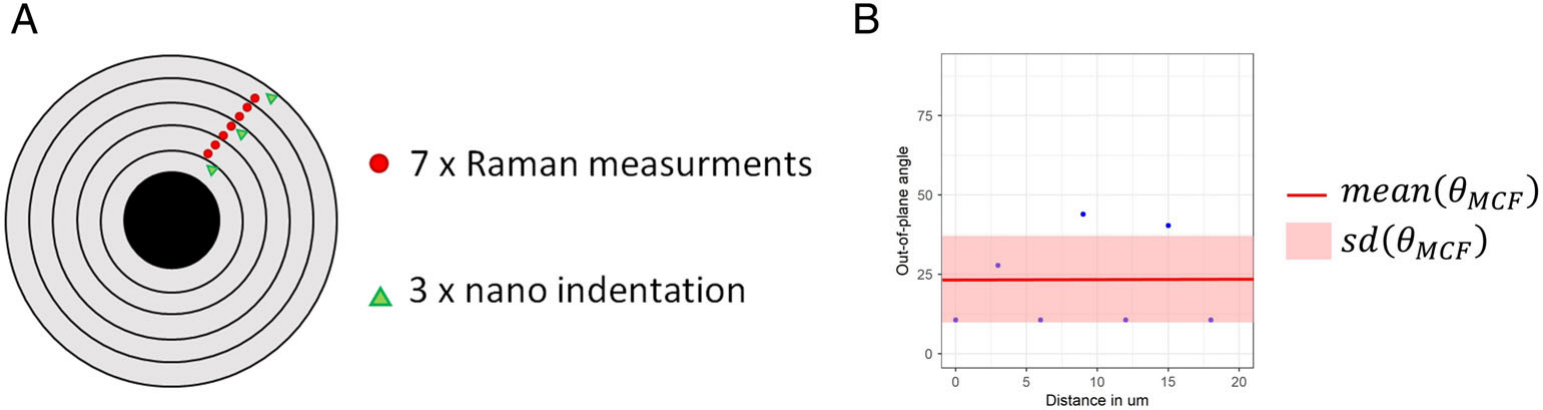
(*A*) Schematic overview of the seven quantitative polarized Ramen spectroscopy (qPRS) measurements arranged in a line‐scan and the site‐matched nanoindentation. (*B*) Representative example of the mineralized collagen fiber (MCF) out‐of‐plane angle alteration within one line scan along the osteon radius to compute the mean (red line) and standard deviation (red box). Measured angle (blue dots).

The qPRS measurements were performed in the same 23 osteonal regions as for the microtensile specimen (healthy control *n* = 8, OI type I *n* = 7, and OI type III *n* = 8). Per osteon, seven qPRS measurements were collected with a 3 μm distance in between, resulting in a line‐scan of 18 μm (see Fig. 3*A*). At each position of the qPRS measurement, 13 Raman spectra, each integrated over 10 seconds, were collected at increasing polarization angles of incoming laser excitation from 0 degrees to 180 degrees with a 15‐degree step. The qPRS sampling volume was ~1.1 μm^3^, with ~0.4 μm in axial and ~1.7 μm in lateral directions, as calculated from the confocal Rayleigh criteria.^[^ ^34^ ^]^ Spectra processing was done in Python v3.8, each spectrum was baseline corrected (second‐order polynomial fit for local minima), and bands of interest were further fit with the Lorentzian function superposition using a least square scheme (scipy.optimize.leastsq). Further details about the background subtraction and peak‐fitting steps can be found elsewhere.^[^ ^25^, ^35^ ^)^ Representative background corrected Raman spectra for each biopsy can be found in the Supporting Information (Fig. S4). The out‐of‐plane orientation of the MCFs, θ_MCF,_ was estimated through the integrated area ratio of amide I/amide III Raman bands following the calibration function proposed by Kochetkova and colleagues.^[^ ^25^ ^]^ For this, the amide I band (1550–1750 cm^−1^, sub‐peaks at ~1638 and 1670 cm^−1^, polarization‐dependent) was normalized over the amide III bands (1215–1300 cm^−1^, sub‐peaks at 1242 and 1273 cm^−1^, polarization‐independent) for all laser polarizations.^[^ ^25^ ^]^ Additionally, the degree of bone mineralization (DBM) was computed as a mineral‐to‐matrix ratio of integrated areas of v_1_PO_4_/amide I Raman bands.^[^ ^36^ ^]^ The ratio of the primary phosphate v_1_PO_4_ Raman band (920–990 cm^−1^, peak at ~960 cm^−1^, polarization‐dependent) over the amide I band was averaged over 13 laser polarization angles to countervail the polarization dependency of the bands. The polarization‐independent mineral‐to‐matrix ratio (v_2_PO_4_/Amide III) could not be extracted because the background noise near the zero shifts of the used Raman system corrupted the second phosphate peak. Finally, using the seven qPRS measurements, a mean and standard deviation of the θ_MCF_ and DBM per osteon were computed (see Fig. 3*B*). The mean value represents the average orientation over three to four lamellas, and the standard deviation indicates the variations within the lamellas.

### Nanoindentation measurements

In the same osteonal region as for the qPRS measurements, three site‐matched nanoindentations were performed (see Fig. 3*A*). The distance between two indents was 7 μm to prevent deleterious artifacts.^[^ ^19^ ^]^ Nanoindentation was conducted with a Berkovic diamond tip mounted on a nanohardness tester (Ultra Nano Hardness Tester; CSM Instruments, Peseux, Switzerland). A trapezoidal loading protocol was used as described in previous work.^[^ ^20^, ^27^ ^]^ First, the tip was lowered with a force rate of 100 mN/min to a final depth of 1 μm. Once this depth was reached, the tip was kept for 30 seconds at this position. Last, the tip was unloaded with a force rate of 400 mN/min. Indentation modulus (E_ind_ [GPa]), hardness (H_IT_ [MPa]), elastic (W_el_ [pJ]), and total (W_tot_ [pJ]) work were extracted.^[^ ^19^, ^37^ ^]^ Finally, the properties of the three nanoindentations were averaged over each of the 23 osteonal regions (healthy control *n* = 8, OI type I *n* = 7, and OI type III *n* = 8).

### Indentation modeling

The indentation modulus is dependent on two main parameters, the orientation of the MCFs (θ_MCF_)^[^ ^33^ ^]^ and DBM.^[^ ^27^ ^]^ Because the properties of healthy human bone in compression are well known,^[^ ^33^ ^]^ the indentation modulus of a healthy control sample could be computed for each θ_MCF_. Therefore, first, a transverse isotropic stiffness tensor was assembled. With this stiffness tensor, indentation moduli were computed at different directions from 0 to 90 degrees out‐of‐plane angle using medtool (v3.7; Dr. Pahr Ingenieurs e.U., Pfaffstätten, Austria). Last, an indentation modulus function $E_{0}^{\mathrm{ind}}\left(\Theta_{\mathrm{MCF}}\right)$ is fitted in the computed indentation moduli, which depends on the MCF orientation. $E_{0}^{\mathrm{ind}}\left(\Theta_{\mathrm{MCF}}\right)$ can be written as a function of $E_{11}$, $E_{33}$, and a shape function $f\left(\theta \right)$ representing the shape of the transverse isotropic indentation tensor (Eq. 3).

(3) $$E_{0}^{\mathrm{ind}}\left(\Theta_{\mathrm{MCF}}\right)=E_{11}+\left(E_{33}-E_{11}\right)f\left(\theta_{\mathrm{MCF}}\right)=E_{11}+\Delta \mathrm{Ef}\left(\theta_{\mathrm{MCF}}\right)$$

Additionally, the indentation modulus depends linearly on the mineralization.^[^ ^27^ ^]^

(4) $$E^{\mathrm{ind}}\left(\mathrm{DBM}\right)=\alpha +\text{βDBM},$$

Equations 3 and 4 were combined into Eq. 5.

(5) $$E^{\mathrm{ind}}\left(\mathrm{DBM},\Theta_{\mathrm{MCF}}\right)=E_{0}^{\mathrm{ind}}\left(\Theta_{\mathrm{MCF}}\right)*E^{\mathrm{ind}}\left(\mathrm{DBM}\right)={\mathrm{\alpha E}}_{11}+\alpha \Delta \mathrm{Ef}\left(\theta_{\mathrm{MCF}}\right)+\beta E_{11}\mathrm{DBM}+\beta \Delta \mathrm{Ef}\left(\theta_{\mathrm{MCF}}\right)\mathrm{DBM}$$

The interaction between $f\left(\theta \right)$ and DBM did not appear significant for our data. Therefore, the model was simplified (Eq. 6).

(6) $$E^{\mathrm{ind}}\left(\mathrm{DBM},\Theta_{\mathrm{MCF}}\right)=E_{0}^{\mathrm{ind}}\left(\Theta_{\mathrm{MCF}}\right)+E^{\mathrm{ind}}\left(\mathrm{DBM}\right)=\left(\alpha +E_{11}\right)+\text{βDBM}+\Delta \mathrm{Ef}\left(\theta_{\mathrm{MCF}}\right)$$

### Statistics

All statistical analyses were performed in R (R version 3.6.0). The measurements were repeated within the same biopsy. Therefore, a mixed‐effect model was used to detect significant differences within the groups (fixed effect) including the biopsies as random effect to reduce the intra variability. The model (Eq. 7) was fitted using the lmer library in R.

(7) $$\text{variabl}\sim \text{Group}+\left(1|\text{biopsy}\right)$$

Difference between the groups were analyzed using a likelihood ratio test between the models with and without the fixed effect. The differences within the groups were evaluated by multiple pairwise comparisons (*pwc*) post hoc test using Dunn's test with a Bonferroni‐Holm *p* value adjustment.

Simple multilinear regression models were used to fit the models to predict the mechanical properties. Adjusted *R* ^2^ was computed, and F‐statistic was applied to check the quality of the fit. The level of significance was set to 95% (*p* < 0.05).

## Results

The three transiliac crest biopsies (healthy control *n* = 1, OI type I *n* = 1, and OI type II *n* = 1) were analyzed with four different methods (μCT, microtensile experiment, quantitative polar Raman spectroscopy [qPRS], and nanoindentation). The results are summarized in Table 1 and the detailed results are stated in the Results sections below.

**Table 1:** Mean and Standard Deviation of the Analyzed Parameters

Method	Parameters	Likelihood ratio test	Healthy control	OI type I	OI type III
Microtensile	σ_ult_ [MPa]	***p* = 2.1e−02**	242 ± 53	359 ± 111	247 ± 25
	ε_ult_ [−]	*p* = 2.4e−01	0.022 ± 0.004	0.023 ± 0.005	0.020 ± 0.002
	E_loading_ [GPa]	***p* = 1.8e−02**	14.8 ± 1.8	20.4 ± 4.4^a^	16.7 ± 1.7
Nanoindentation	E_ind_ [GPa]	***p* = 1.2e−04**	17.2 ± 2.2	24.9 ± 1.5^a^	21.9 ± 0.6^a^
	H_it_ [MPa]	***p* = 1.4e−04**	524 ± 49	907 ± 137^a^	758 ± 33^a^
	W_el_ [pJ]	***p* = 3.3e−04**	1149 ± 159	1952 ± 348^a^	1727 ± 183^a^
	W_tot_ [pJ]	***p* = 1.9e−04**	5633 ± 478	8591 ± 1128^a^	7538 ± 392^a^
μCT	TMD [mgHA/cm^3^]	N/A	869.8	989.9	979.9
Raman spectroscopy	v_1_PO_4_/amide I [−]	***p* = 4.8e−02**	2.60 ± 0.63	3.12 ± 0.26	3.22 ± 0.19
	Θ_MCF_ [°]	*p* = 7.4e−02	39.4 ± 14.3	30.3 ± 6.3	41.8 ± 5.4^b^
	sd Θ_MCF_ [°]	*p* = 6.4e−01	10.6 ± 3.6	11.9 ± 4.1	12.4 ± 4.7

*Note*: Each group contains one biopsy (healthy control *n* = 1, OI type I *n* = 1 and OI type II *n* = 1). The number of experiments within the group for microtensile experiments (ultimate strength σ_ult_, ultimate strain ε_ult_, and loading modulus E_loading_), nanoindentation (indentation modulus E_ind_, hardness H_it_, elastic W_el_, and total work W_tot_), and qPRS (degree of mineralization v_1_PO_4_/amide I, mean out‐of‐plane agnle Θ_MCF_, and standard deviation of out‐of plane angle sd Θ_MCF_) are *n* = 8, *n* = 7, and *n* = 8 for healthy control, OI type I, and OI type III, respectively. TMD contains only one value per sample and therefore no statistic. TMD results were already published in.^[^ ^27^ ^]^ Values of *p* were computed using the likelihood ratio test. Bold indicates statistically significant.

Abbreviation: TMD = tissue mineral density.

^a^ Groups that are significantly different to healthy control (Dunn's test).

^b^ Variables that are significantly different between the OI types (Dunn's test).

### Global mineralization

The global mineralization, measured with μCT, was in both OI biopsies approximately 100 mg HA/cm^3^ higher (OI type I and type III are 14% and 13% higher, respectively; see Table 1). Statistical tests for differences in TMD were not performed due to the low sample size.

### Microtensile experiments

**Fig. 4:**
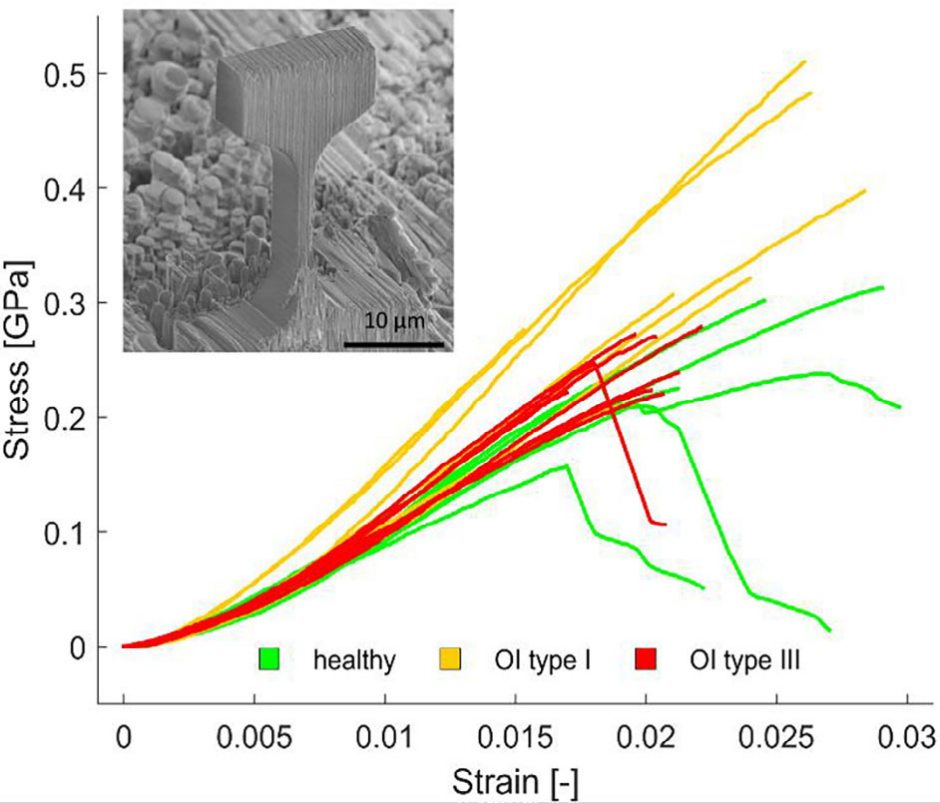
Stress–strain curves of all microtensile experiments.

**Fig. 5:**
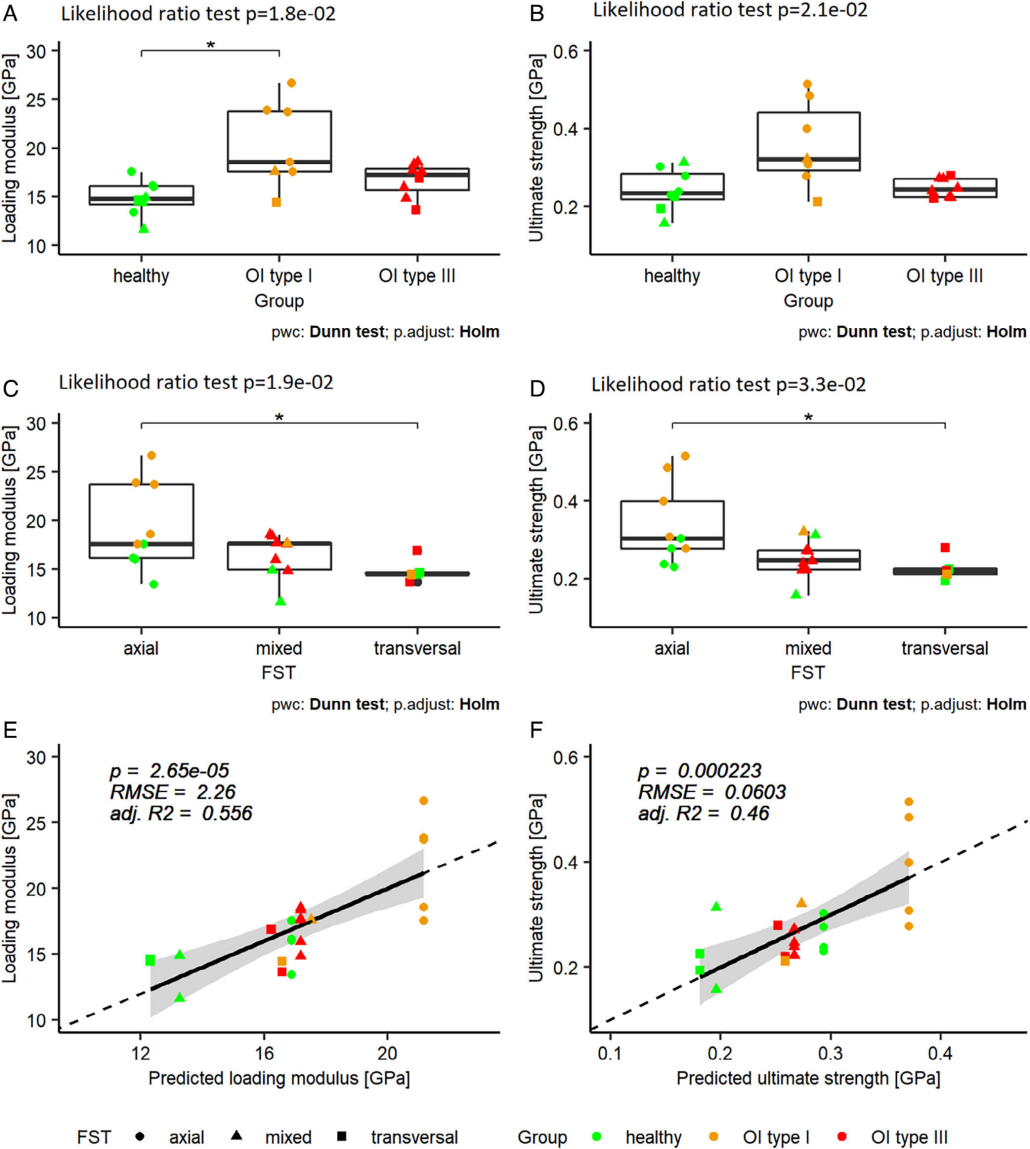
Loading modulus (*A*,*C*,*E*) and ultimate strength (*B*,*D*,*F*) versus the groups, the fracture surface, and the predicted values. For *E* and *F*, the values were predicted with Eqs. 1 and 2, respectively.

Qualitative investigation revealed that most of the microtensile specimens failed in a brittle manner (see Fig. 4). However, three of eight healthy control specimens and one of eight OI type III specimens yielded and exhibited a post‐yield behavior. The maximum strain was between 12% and 31% higher than the ultimate strain for those four specimens. All OI type I specimens had brittle behavior. The likelihood ratio test revealed a significant difference among the groups in loading modulus (*p* = 1.8e−02) and ultimate strength (*p* = 1.8e−02). Loading modulus was the highest in OI type I and significantly different than healthy control (*p* = 1.2e−02, see Fig. 5*A*). Similar to loading modulus, the highest mean ultimate strength was observed in OI type I, but the *pwc* did not detect any significant difference among the groups (see Fig. 5*B*). Interestingly, strain to failure is independent among the groups. The ultimate strength is positively and significantly correlated (*R* ^2^ = 0.60, *p* = 8.5e−06) to the loading modulus (E_loading_).

**Fig. 6:**
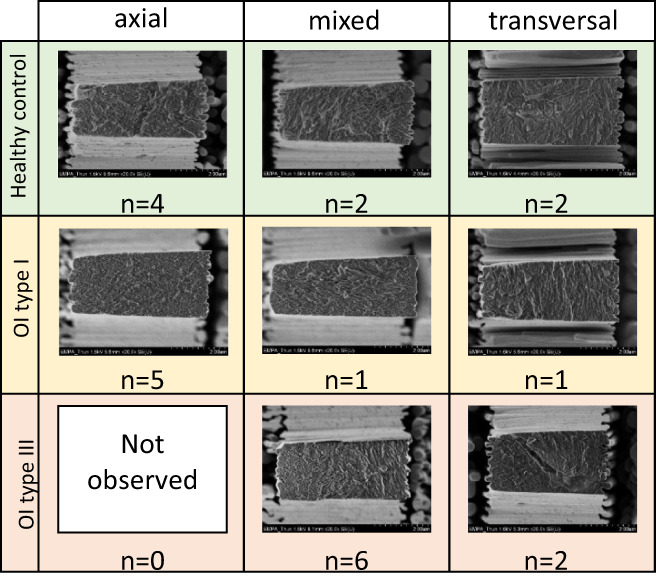
Representative fracture surface for each biopsy and each FST.

All three FSTs were found in the biopsies. However, the FSTs were unevenly distributed among the biopsies (see Fig. 6). The healthy control biopsy revealed four axial, two mixed, and two transversal FSTs. On the other hand, the OI type I biopsy presented five axial, one mixed, and one transversal FST. However, in the OI type III biopsy, six mixed and two transversal FST were found, and no axial FST. In all three biopsies, voids (eg, canaliculi) were identified as stress concentrators. Voids were present in four healthy, two in OI type I, and three in OI type III microtensile specimens. SEM fracture surface images of each tensile specimen are provided in the Supporting Information (healthy control: Fig. S1, OI type I: Fig. S2, and OI type III: Fig. S3). The *pwc* of the FST for the loading modulus and the ultimate strength revealed that specimens with axial FST have significantly higher properties than transversal FST specimens (for loading modulus: *p* = 1.9e−02 see Fig. 5*C*, and for ultimate strength: *p* = 3.3e−02 see Fig. 5*D*). FST and TMD are both significant factors to predict the loading modulus and the ultimate strength. with an adjusted *R* ^2^ of 0.556 (*p* = 2.7e−05) and 0.46 (*p* = 2.2e−04), respectively (see Fig. 5*E,F*).

### Site‐matched nanoindentation and quantitative polarized Raman spectroscopy

The mean out‐of‐plane angle of the MCF of the line‐scan was significantly different among the groups (Likelihood ratio test, *p* = 4.8e−02). Healthy control samples have a more considerable variation of mean out‐of‐plane angle. Furthermore, the *pwc* revealed that the mean out‐of‐plane angle in OI type I was significantly more aligned in the longitudinal direction than OI type III. Nevertheless, the standard deviation of the line‐scan, a measurement for the variation between lamellas, was not significantly different among the groups and was approximately 11 degrees. Furthermore, the local level of mineralization is not significantly different among the groups. However, there is a trend that OI bone has higher mineralization than healthy bone (see Table 1).

**Fig. 7:**
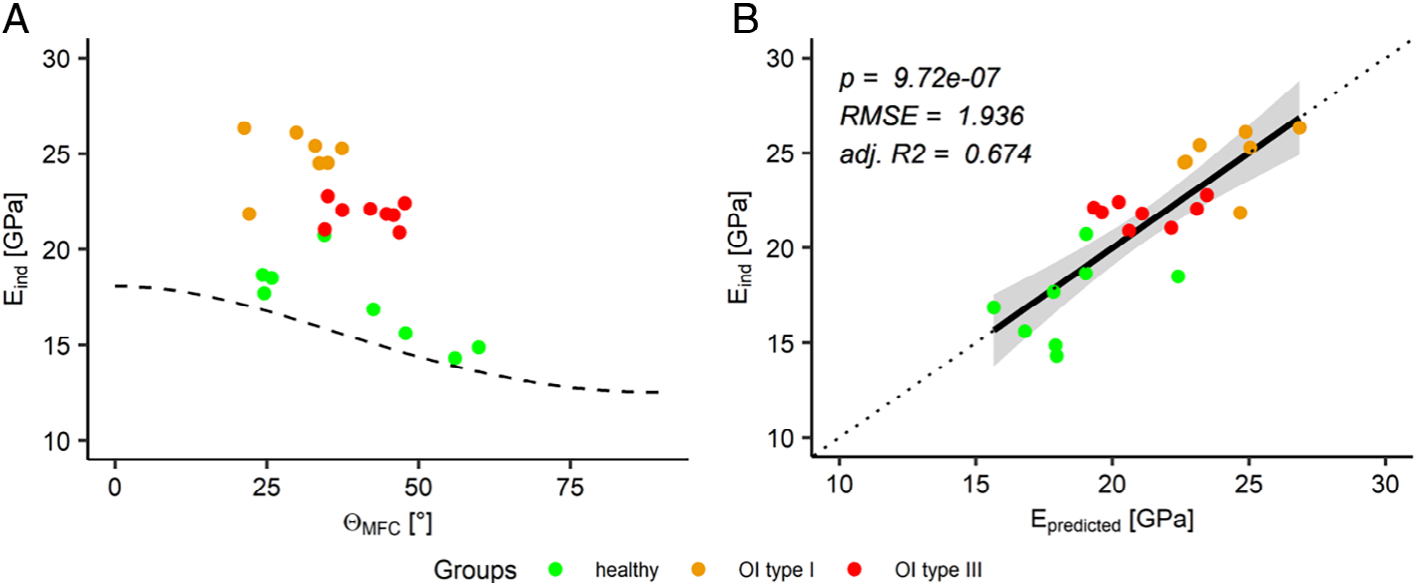
(*A*) Indentation modulus versus out‐of‐plane angle of the mineralized collagen fiber ($\theta_{\mathrm{MCF}}$), black dashed line shows the indentation modulus from the transverse isotropic indentation tensor computed with the parameters reported.^[^ ^33^ ^]^ (*B*) Indentation modulus versus the predicted indentation modulus (Eq. 6).

All the analyzed indentation properties (indentation modulus, hardness, elastic and plastic work) are significantly higher in OI biopsies compared to healthy control (see Table 1). Furthermore, the indentation modulus is highly dependent on the orientation of the collagen fibers (*p* = 2.13e−05, see Fig. 7*A*) in the multilinear model. However, Fig. 7*A* indicates an offset among the three different biopsies. This offset can be compensated by including DBM (*p* = 5.23e−06) into the model. The indentation model predicts the modulus with the information of the MCF orientation and the DBM with an *R* ^2^ of 67.4% (*p* = 9.72e−07, see Fig. 7*B*).

## Discussion

This study intended to capture the mechanical behavior of two different OI types at the ECM level in tension and to compare it to healthy bone ECM. To do this, microtensile specimens (*n* = 23) were fabricated from three biopsies (control *n* = 1, OI type I *n* = 1, and OI type III *n* = 1), loaded under tension and mechanical properties were extracted. Interestingly, the mechanical properties of dry OI ECM were not inferior compared to healthy control. Furthermore, the relationship among the measured tensile properties, the degree of mineralization, and angular orientation of the MCF were established. Those relationships were supported by nanoindentation and site‐matched qPRS measurements. For the small sample size, the mechanical properties, acquired with both methods, were mostly consistent dependent on the degree of mineralization and the orientation of the MCFs. OI biopsies were collected from individuals who underwent a bisphosphonate treatment for approximately 5 years during growth. Weber and colleagues^[^ ^9^ ^]^ reported no significant change of the indentation properties and mineralization after 2.5 year bisphosphonate treatment in children. Nevertheless, the presented properties in this study are typical for 5‐year bisphosphonate‐treated individuals. In the remainder of this section, the results are discussed in more detail.

### Degree of mineralization

The degree of mineralization was analyzed with two different methods. First, the global mineralization was measured with a hydroxyapatite calibrated μCT, which directly measures the mass of bone mineral in a given volume. The second method was through the mineral‐to‐matrix ratio, assessed via qPRS. The mineral‐to‐matrix ratio is extracted from the Raman spectrum, providing a relative value for the degree of mineralization and is frequently used to describe the bone matrix mineralization.^[^ ^38^ ^]^ The mineral‐to‐matrix ratio is limited to a relative amount of minerals over collagen. However, both methods (μCT and qPRS) revealed the same trend of higher mineralization levels in OI bone compared to healthy control and are in agreement with values reported in various studies.^[^ ^7^, ^8^ ^]^

### Orientation of mineralized collagen fibers

Orientation of the mineralized collagen fibers was investigated with two different methods. First, the MCF orientation of the tensile specimen was qualitatively analyzed by categorizing the FST into axial, mixed, and transversal. The FSTs in healthy were almost equally distributed. On the other hand, in OI type I, the FSTs were primarily oriented in the axial direction and in OI type III in transversal and mixed. The second method to quantify the MCF orientation was done by qPRS, and similar trends were found. In general, the healthy biopsy has a more considerable variation in MCF orientation than both OI biopsies. However, a general statement cannot be made because both measurements analyze the MCF orientation punctually in a small testing region. Additionally, the ilium is an irregular bone, and the orientation of osteons and their lamellas may not be perfectly aligned.

### Mechanical properties

#### Microtensile properties

In vacuo/dry testing condition at the ECM level revealed an average ultimate strength of 242 ± 53 MPa at a strain of 2.2% ± 0.4% for healthy human bone ECM. The loading modulus was 14.8 ± 1.82 GPa. To our best knowledge, this is the first study reporting tensile properties at this length scale for human bone tissue. The reported values in the presented study for the ultimate strength of healthy human control are within the range of the transverse (130 ± 20 MPa) and axial (350 ± 50 MPa) direction of the ovine bone.^[^ ^23^ ^]^ At the macroscopic level, dry human diaphyseal bone has an elastic modulus and tensile ultimate strength of 18.5 ± 2.9 GPa and 117.8 ± 27.1 MPa, respectively.^[^ ^39^ ^]^ These values were often found to be lower when the cortex becomes thinner as in the cortical shell of the femoral neck^[^ ^40^ ^]^ or the vertebral body.^[^ ^19^ ^]^ The measured elastic modulus at the macroscopic level is therefore close to the loading modulus at the tissue level for thin cortices. However, the loading modulus was defined as the highest slope of the loading curve and is not equivalent to the elastic modulus. On the other hand, the ultimate strength is two times smaller at the macroscopic level. Vascular pores and interfaces, which are more present in larger volumes, act as potential stress concentration and increase the likelihood of crack initiation.

Interestingly, the tensile properties of the OI bone were not inferior compared to the healthy control. The strain to failure was similar among the groups. However, OI type I biopsy revealed a higher loading modulus and ultimate strength than healthy control and OI type III bone. Independent on the disease type, the ultimate tensile strength of the bone ECM is highly dependent on the stiffness (*R* ^2^ = 0.60, *p* = 8.5e−06). This indicates that stiffer bone also has a higher strength in dry conditions.

Four tensile specimens (control *n* = 3 and OI type III *n* = 1) revealed some post‐yield behavior. The FSTs for those samples were axial or mixed, meaning that there were for all four specimens fully or partial axial MCFs alignment. On the other hand, no yielding was observed in the transversal FSTs. Therefore, there is a potential toughening mechanism, when the MFCs are pulled out of the extrafibrillar matrix. Still, most of the axial and mixed samples failed in a fully brittle manner.

#### Microtensile properties versus MCF orientation and TMD

In compression, the mechanical properties are mainly dependent on the degree of mineralization^[^ ^27^ ^]^ and orientation of the MCFs.^[^ ^20^ ^]^ The direction of the MCF alignment in the microtensile experiment is based on three classes according to its FST (axial, mixed, and transverse). Loading modulus and ultimate strength showed an anisotropic behavior with higher axial direction properties than the transverse direction, which agrees with data reported in ovine bone.^[^ ^23^ ^]^

It has been shown that mineralization increases the stiffness in compression.^[^ ^27^ ^]^ Because the relative changes between the two loading modes (compression and tension) in the elastic regime are negligible (see Table 2), the mineralization should also increase the stiffness in tension. The simple multilinear model indicated that the loading modulus (Eq. 1) and the ultimate strength (Eq. 2) are both dependent on the FST and the TMD.

**Table 2:** Comparison of Micromechanical Properties Between Compression^[^ ^27^ ^]^ and Tension

Property	Biopsy	Tension	Compression	Relative change [%]
Strength [MPa]	Healthy control	242 ± 53 (*n* = 8, ultimate)	584 ± 18 (*n* = 2, ultimate) 359 ± 31 (*n* = 2, yield)	−58.6 −32.6
	OI type I	359 ± 111 (*n* = 7, ultimate)	753 ± 53 (*n* = 7, ultimate) 455 ± 61 (*n* = 7, yield)	−52.3 −21.1
	OI type III	247 ± 25 (*n* = 8, ultimate)	770 ± 121 (*n* = 15, ultimate) 397 ± 86 (*n* = 15, yield)	−67.9 −37.8
Strain [−]	Healthy control	0.022 ± 0.004 (*n* = 8, ultimate)	0.067 ± 0.009 (*n* = 2, ultimate) 0.023 ± 0.003 (*n* = 2, yield)	−67.2 −4.3
	OI type I	0.023 ± 0.005 (*n* = 7, ultimate)	0.078 ± 0.015 (*n* = 7, ultimate) 0.028 ± 0.002 (*n* = 7, yield)	−70.5 −17.8
	OI type III	0.020 ± 0.002 (*n* = 8, ultimate)	0.142 ± 0.047 (*n* = 15, ultimate) 0.024 ± 0.002 (*n* = 15, yield)	−85.9 −16.7
Loading modulus [GPa]	Healthy control	14.8 ± 1.82 (*n* = 8)	16.8 ± 0.71 (*n* = 2)	−13.7
	OI type I	20.4 ± 4.42 (*n* = 7)	17.6 ± 1.94 (*n* = 7)	15.9
	OI type III	16.7 ± 1.74 (*n* = 8)	17.9 ± 3.76 (*n* = 8)	−6.7

#### Indentation modulus versus MCF orientation and DBM

Unfortunately, site‐matched qPRS could not be performed on the fracture surface of the tensile specimen. Therefore, additional site‐matched nanoindentations and qPRS measurements were conducted at the same osteons from the tensile tests. The indentation modulus, hardness, elastic, and plastic work were higher for OI than healthy control bone. Furthermore, using the information of the MCF orientation and the degree of mineralization of the site‐matched qPRS measurements, the indentation modulus can be predicted with an *R* ^2^ of 67.4%. The model indicates that influence of the MCFs orientation (*p* = 5.22e−06) is higher than the degree of mineralization (*p* = 2.13e−05). Indermaur and colleagues^[^ ^27^ ^]^ reported that the indentation modulus is dependent on the mineralization with an R^2^ of 31%. Therefore, including the orientation of the MCFs in the mineralization model helps to better predict the indentation modulus.

#### Comparison between tensile and compression micromechanical properties

Micropillar compression tests were performed at the ECM level on the same three biopsies,^[^ ^27^ ^]^ allowing the comparison between the two different loading modes. However, only trends could be shown because the number of tested samples between the two modes is unbalanced, and the testing region is different (different osteonal regions). The ultimate strength was, on average, reduced by 50% in tension compared to compression. This enormous difference between the two loading modes can be explained by the different post‐yield behavior. Bone ECM in compression revealed a distinct post‐yield behavior with softening in the axial direction and hardening in the transversal direction.^[^ ^20^ ^]^ On the other hand, bone under tension shows a brittle behavior and fails rapidly after yielding, except for four specimens. Therefore, the ultimate tensile strength should be compared with the compressive yield strength, which was defined by the 0.2% offset rule. The tensile ultimate strength is approximately 20% lower than compression yield strength. As expected from a previous study in ovine bone ECM^[^ ^23^ ^]^ and hard tissues in general, a similar loading modulus was observed in compression and in tension. Interestingly, similar relative differences in ultimate strength between tension and compression were observed in ovine bone.^[^ ^23^ ^]^

### Limitations

This study is limited by the low number of biopsies and, to a lower extent, by the number of specimens within the biopsies. In fact, the study comprises only 23 microtensile specimens in total (healthy control *n* = 8, OI type I *n* = 7, OI type III *n* = 8), but represents some 138 hours of fabrication. The reported difference among the groups may be influenced by the variation of the subjects and not only by the disease. The small sample size also limits the statistics, and more data is needed to claim a universal model. Nevertheless, the authors believe that the presented data are consistent and relevant for a better understanding of the mechanical behavior of bone at the ECM level.

Another limitation is the in vacuo/dry testing conditions that are different from the physiological one. Wet bone tissue has a lower modulus, lower strength but a more ductile response, and additional toughening mechanisms are present.^[^ ^24^ ^]^ However, at this scale, the rehydrated bone ECM may also be subject to artifacts such as anisotropic swelling due to unnatural interfaces created during in vacuo manufacturing. At higher level of organization, it has been shown that the wet OI bone tissue revealed less toughness and lower ultimate strength in OI mice models^[^ ^41^, ^42^, ^43^, ^44^, ^45^ ^]^ compared to controls. A potential explanation why this is not present at the ECM level might be the lack of larger voids and interfaces (eg, higher cortical porosity) which reduces toughness. On the other hand, we cannot completely deny that the mechanical behavior in rehydrated/wet condition might reveal different behavior between healthy/control and OI bone ECM. Nevertheless, the testing conditions can be justified by the comparative nature of the study, but it cannot be completely excluded that existing mechanical differences in wet condition may disappear in the dry state and require additional experiments in wet testing conditions.

Furthermore, tissue fixation and PMMA infiltration may artificially change the behavior of human bone ECM in tension. Rodriguez‐Florez and colleagues^[^ ^46^ ^]^ showed that microhardness is increased in embedding bone specimens with PMMA which may be explained by the PMMA‐filled nanopores. However, in a yet unpublished study of ours, one ovine bone specimen underwent the same fixation protocol (formalin fixation, dehydration with ethanol and PMMA infiltration) as described in the materials section and microtensile experiments and unpolarized Raman spectroscopy were performed. Microtensile experiments revealed that the loading modulus and ultimate strength of fixed‐air‐dried ovine bone ECM were not significantly different compared to published native‐dry ovine bone ECM.^[^ ^24^ ^]^ Furthermore, mineral‐to‐matrix ratio using Raman spectroscopy was not significantly changed by the tissue fixation and PMMA infiltration. On the other hand, the three human biopsies in this study were reused from two previous studies (healthy/control biopsy of Glorieux and colleagues in 2000^[^ ^31^ ^]^ and OI biopsies of Rauch and colleagues in 2006^[^ ^32^ ^]^). PMMA properties may change due to the physical aging (storage temperature and environmental condition). Even it has been reported that PMMA at 20°C has a lifetime of 25 years,^[^ ^47^ ^]^ the age of the biopsies may have a different influence on the properties compared to the tested ovine biopsy. Furthermore, tissue fixation may affect the properties of human bone differently than ovine bone.

Another limitation is that the tensile model contains only discrete factors. The anisotropy values for the tensile specimen were determined by three distinct FSTs. Additionally, only a global mineralization value was available per biopsy. Therefore, an additional indentation model was developed to validate the assumption for the angular and mineralization dependencies of the tensile properties.

Last, site‐matched measurements are essential to building complex models in a heterogeneous material such as bone. However, the testing region between two methods can never be precisely at the same position leading to uncertainty and bias.

## Conclusion

This study analyzed three transiliac biopsies from two OI patients and a healthy control with four independent methods. Most importantly, tensile properties of both OI bone ECM biopsies were not inferior to those of the healthy control biopsy but followed consistent relationships with degree of mineralization and collagen fiber orientation Together with the same observation for compressive properties in a larger set of OI and control biopsies, we hypothesize that the genetic defects underlying the considered types of OI have a minor impact on the micromechanical properties of bone ECM. Given the limited number of samples and the dry condition of the mechanical tests, further studies will be necessary to refine this unexpected finding and verify if the observed bone fragility in OI can be explained by the dramatic reduction in bone mass or other alterations in bone architecture at a higher scale.

## Author Contributions

**Michael Indermaur:** Conceptualization; data curation; formal analysis; investigation; methodology; writing – original draft; writing – review and editing. **Daniele Casari:** Conceptualization; formal analysis; investigation; methodology; writing – review and editing. **Tatiana Kochetkova:** Formal analysis; funding acquisition; investigation; methodology; software; writing – review and editing. **Bettina M. Willie:** Resources; writing – review and editing. **Johann Michler:** Resources; writing – review and editing. **Jakob Schwiedrzik:** Conceptualization; methodology; resources; writing – original draft; writing – review and editing. **Philippe Zysset:** Conceptualization; formal analysis; methodology; project administration; resources; supervision; writing – original draft; writing – review and editing.

## Disclosures

All authors have nothing to disclose and no conflict of interest.

### Peer Review

The peer review history for this article is available at https://www.webofscience.com/api/gateway/wos/peer-review/10.1002/jbm4.10826.

## Supporting information


**Data S1:** Supplementary Information

## Data Availability

Data available on request from the authors.
