# Supplementary material for: Tensile Mechanical Properties of Dry Cortical Bone Extracellular Matrix: A Comparison Among Two Osteogenesis Imperfecta and One Healthy Control Iliac Crest Biopsies
Source: JBMR Plus. 2023 Oct 11;7(12):e10826. doi: 10.1002/jbm4.10826 (PMC10731133; doi:10.1002/jbm4.10826)
Supplement: Supplementary file 1 — Data S1: Supplementary Information [file JBM4-7-e10826-s001.pdf]

Tensile mechanical properties of dry cortical bone extracellular matrix: a comparison among two osteogenesis imperfecta and one healthy control iliac crest biopsies

Supplementary materials:

Michael Indermaur* [a)], Daniele Casari* [a,b)], Tatiana Kochetkova [b)], Bettina M. Willie [c)], Johann Michler [b)], Jakob Schwiedrzik [b)], Philippe Zysset [a)]

a) ARTORG Center for Biomedical Engineering, University of Bern, Switzerland
b) Swiss Federal Laboratories for Material Science and Technology, Empa, Thun, Switzerland
c) Research Centre, Shriners Hospital for Children-Canada, Department of Pediatric Surgery, McGill University, Montreal, Canada

* Authors contributed equally to this work

Fracture surface type

Scanning electron microscope images of the tensile specimen were collected before and after mechanical testing (see figure 1,2 and 3). Post-testing images were used to classify the fracture surface type (FST). In figure 1, 2, and 3 you will find the defined FST for each micro tensile specimen which green arrow indicating voids (e.g. canaliculi).

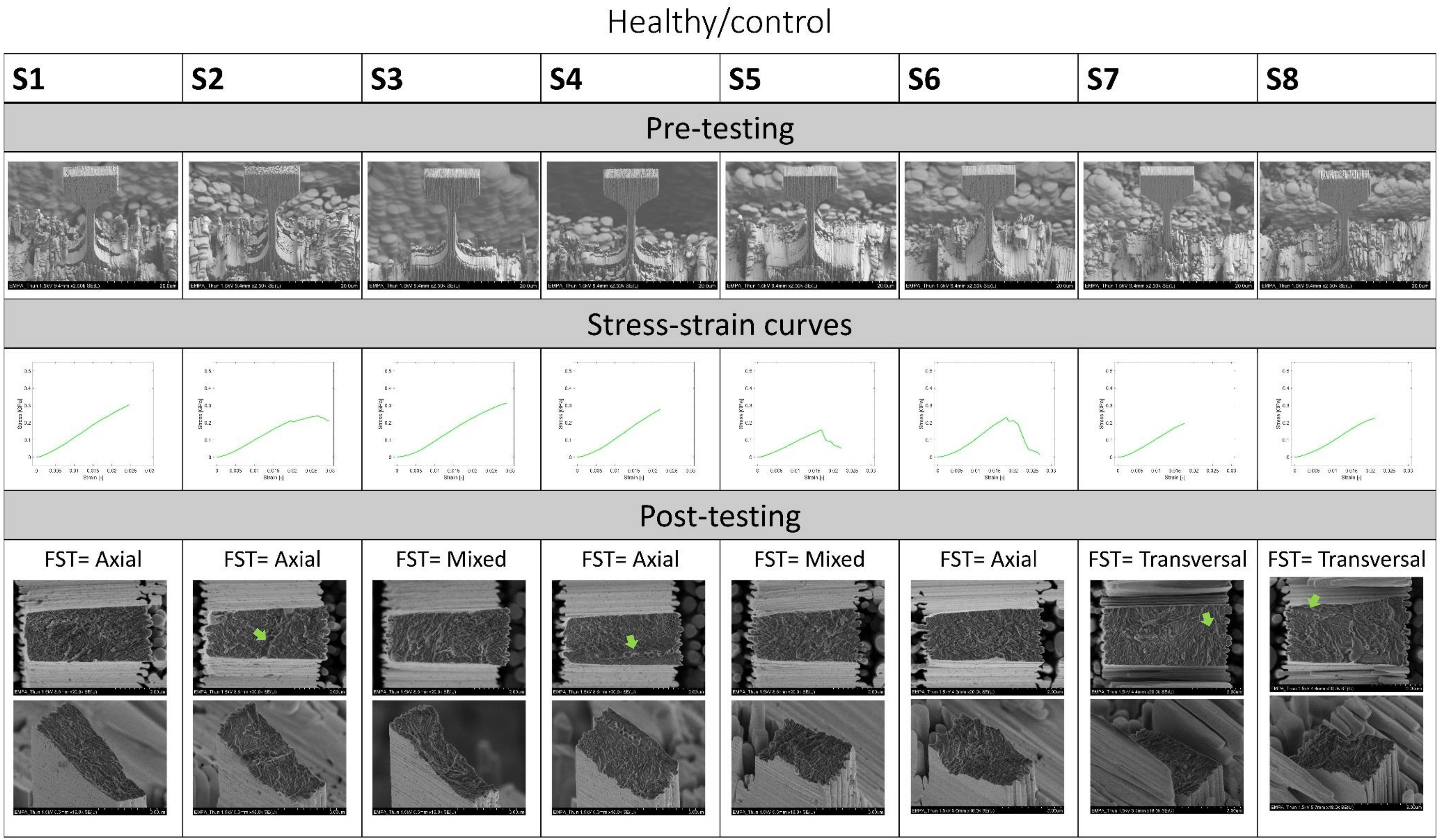

Figure 1: Scanning electron images of the healthy control tensile specimens. Before and after mechanical testing. Post-testing images were used to classify the fracture surface type.

OI type I

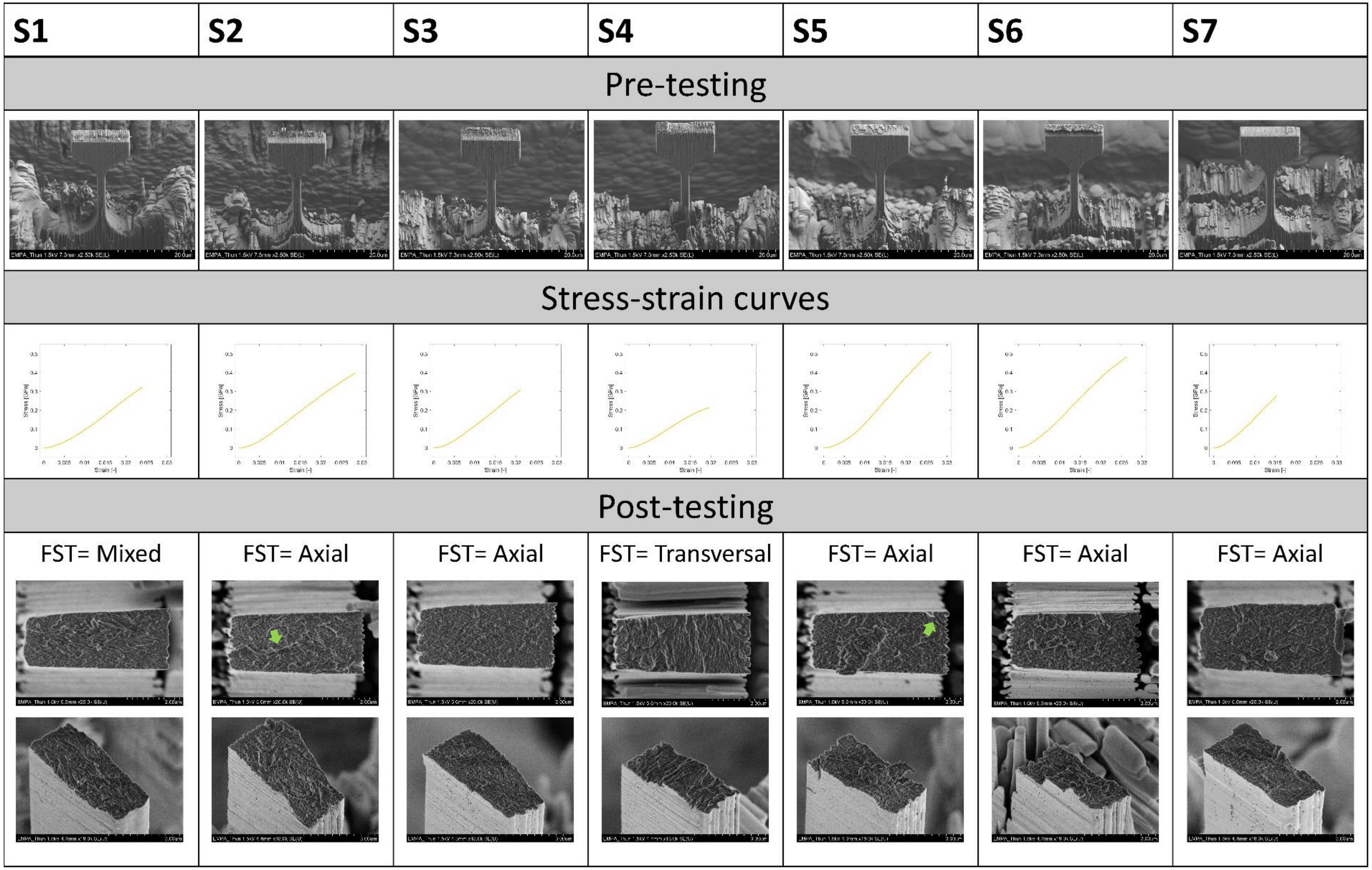

Figure 2: Scanning electron images of the OI type I tensile specimens. Before and after mechanical testing. Post-testing images were used to classify the fracture surface type.

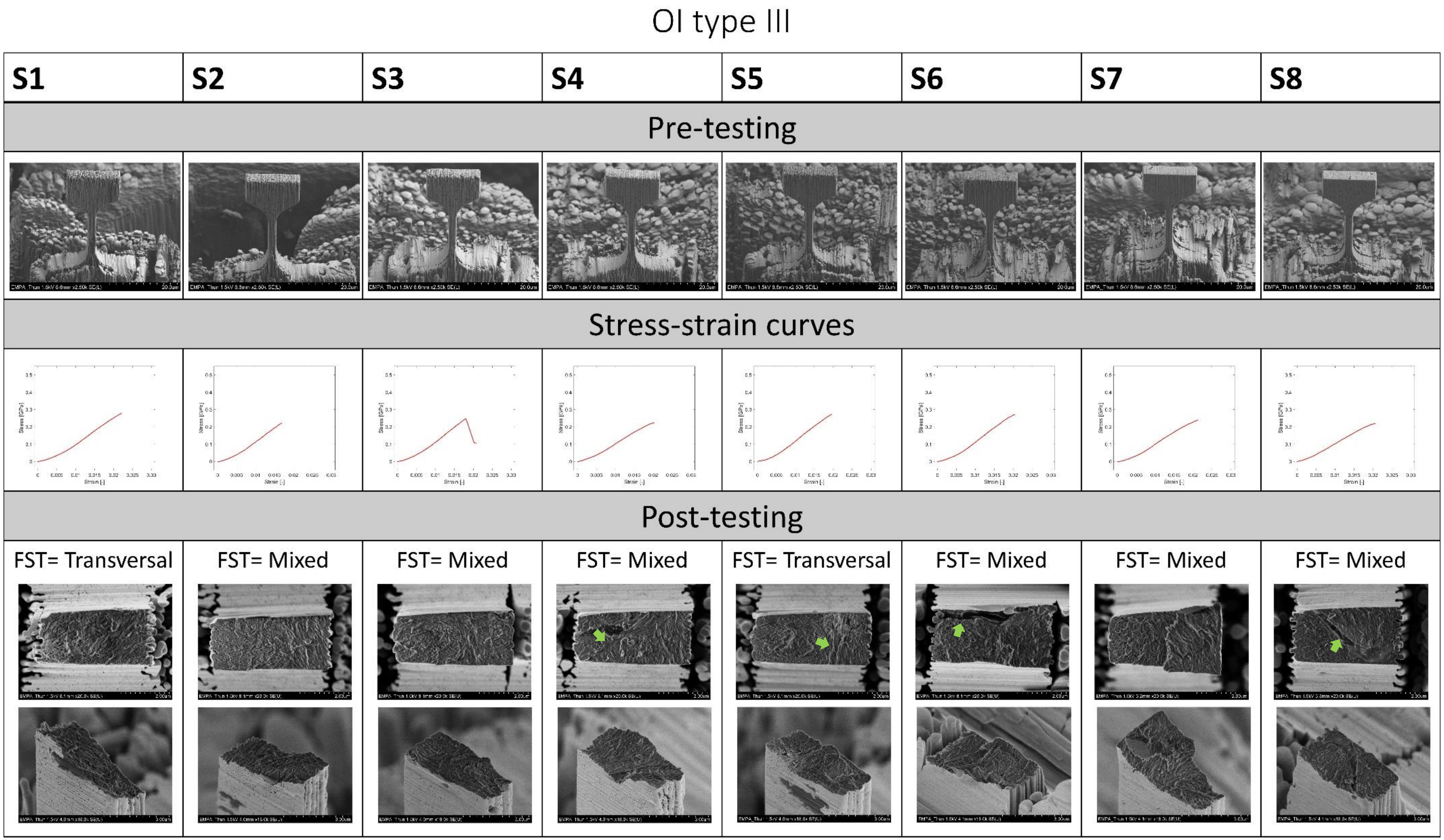

Figure 3: Scanning electron images of the OI type III tensile specimens. Before and after mechanical testing. Post-testing images were used to classify the fracture surface type.

Raman Spectroscopy

Figure 4 shows a representative Raman spectrum for healthy control, OI type I and OI type III specimen. Those spectra were corrected by the background light.

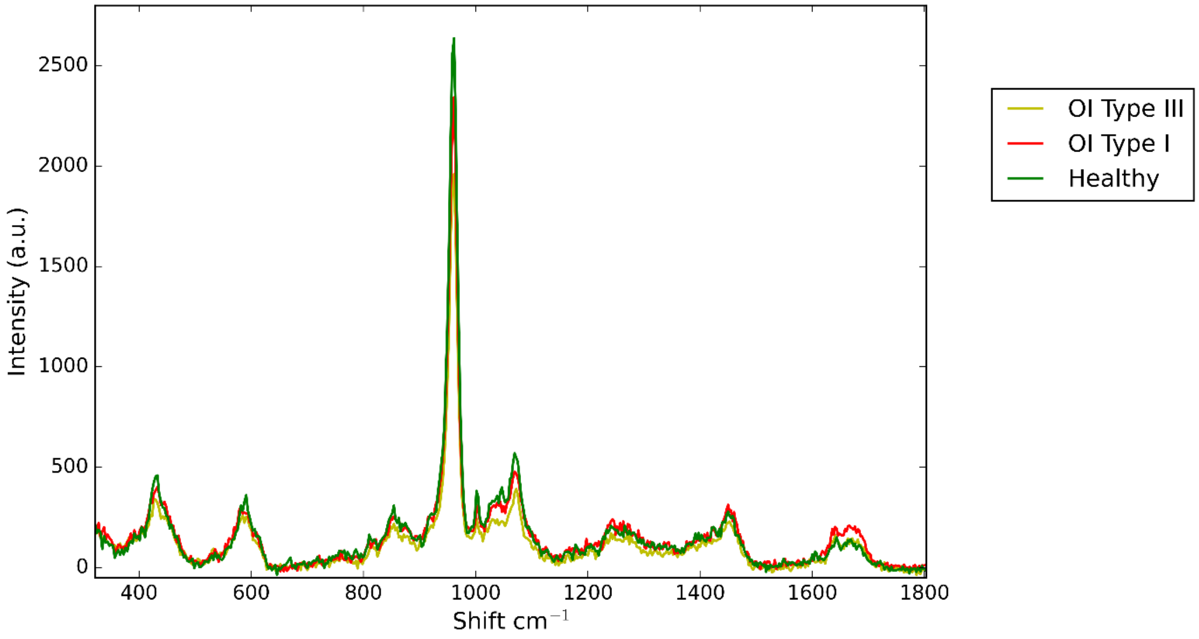

Figure 4:Representative Raman spectrum at polarization angle 0°. Healthy control = green, OI type I in red and OI type III in light green
